# Microalbuminuria and Retinopathy among Hypertensive Nondiabetic Patients at a Large Public Outpatient Clinic in Southwestern Uganda

**DOI:** 10.1155/2018/4802396

**Published:** 2018-06-06

**Authors:** Peter Kangwagye, Joselyn Rwebembera, Tony Wilson, Francis Bajunirwe

**Affiliations:** ^1^Department of Internal Medicine, Mbarara University of Science and Technology, P.O. BOX 1410, Mbarara, Uganda; ^2^Uganda Heart Institute, P.O. BOX 7051, Kampala, Uganda; ^3^Department of Community Health, Mbarara University of Science and Technology, P.O. BOX 1410, Mbarara, Uganda

## Abstract

**Background:**

Routine testing of microalbuminuria and retinopathy is not done among patients with high blood pressure in resource-limited settings. We determined the prevalence of microalbuminuria and retinopathy and their risk factors among hypertensive patients at a large hospital in western Uganda.

**Methods:**

We consecutively recruited nondiabetic patients with hypertension at the outpatients' clinic over a period of 3 months. Spot urine samples were tested for urine albumin. Direct fundoscopy was done to assess retinal vasculature and optic disc for signs of hypertensive retinopathy. Logistic regression was done with retinopathy and microalbuminuria as primary outcomes.

**Results:**

We enrolled 334 patients and, of these, 208 (62.3%) were females, with median age of 55 years (range: 25–90). The prevalence of microalbuminuria was 59.3% (95% CI: 50.1–72.2) and that of retinopathy was 66.8% (95% CI: 58.6–76.5). The independent correlates of retinopathy and microalbuminuria were systolic blood pressure (SBP) > 140 mmHg (OR = 2.76, 95% CI: 1.29–5.93) and treatment with beta-blockers (OR = 2.16, 95% CI: 1.05–4.44). Use of ACEIs was unrelated to the study outcomes.

**Conclusion:**

The prevalence of retinopathy and microalbuminuria is high. Clinicians should aim for better control of blood pressure and routinely perform fundoscopy and urine albumin, especially for patients with poorly controlled blood pressure.

## 1. Introduction

Hypertension is the most common cardiovascular disorder in the world, affecting approximately one billion people globally, and accounts for approximately 7.1 million deaths annually [1]. In sub-Saharan Africa, the incidence of hypertension is on the increase most likely because of changes in lifestyle and diet [2–4] with a high burden of hypertension remaining undiagnosed [5]. In Uganda, a recent community-based nationwide survey has shown that the prevalence of hypertension is consistently over 20% in the general adult population across all regions [6] and is as high as 35% in some urban centers.

Hypertension is a major modifiable risk factor for end organ failure, although the exact level at which these complications start to develop is not completely understood [7]. The kidney and the eyes are two of the end organs that suffer complications from hypertension. In the kidneys, microalbuminuria occurs and is a marker of kidney disease and endothelial dysfunction and predictor of end-stage renal disease and cerebrovascular disease [8, 9]. Studies have found that majority of patients with poorly controlled hypertension have microalbuminuria and that its prevalence increases with the duration and severity of hypertension [10]. In sub-Saharan Africa, majority of hypertensive patients have poorly controlled blood pressure [11–13], yet few studies have been conducted. The few studies suggest that prevalence of microalbuminuria may be as high as 32.3% among hypertensive nondiabetic adults in Nigeria [14] and 39.5% among newly diagnosed hypertensive patients at the national referral hospital in Uganda [15].

Microalbuminuria is predictive of all-cause and cardiovascular disease (CVD) mortality in men and women independently of other established CVD risk factors, with microalbuminuric individuals having approximately a 50% increased risk of all-cause mortality [16–18]. Therefore, microalbuminuria is an important biomarker for renal disease among patients with CVD. Clinical trials suggest that early recognition and reduction of microalbuminuria have not only renal but also cardioprotective benefits [19]. In sub-Saharan Africa, few studies have measured microalbuminuria among nondiabetic hypertensive patients receiving treatment. In the presence of high blood pressure, retinal circulation undergoes a series of pathophysiological changes with a spectrum of signs commonly referred to as hypertensive retinopathy. Like microalbuminuria, hypertensive retinopathy is associated with subclinical cerebrovascular disease and predicts incidental clinical stroke, congestive heart failure, and cerebrovascular mortality, independent of blood pressure and other traditional risk factors [20]. In the Atherosclerosis Risk in Communities (ARIC) study, individuals with retinal hemorrhages and cotton wool spots were 2–4 times more likely to develop an incident clinical stroke within 3 years, even after controlling for blood pressure, smoking, and other risk factors [20]. Majority of these observations have been made not only among non-African populations and in resource-rich settings but also among patients with diabetes [21]. Therefore, we sought to measure the independent effects of hypertension on both microalbuminuria and retinopathy among nondiabetic patients.

The occurrence of kidney and retinal complications may be higher than that observed in studies in the western world. Routine monitoring of microalbuminuria and assessment for retinopathy are not done in limited-resource settings because the clinics are very large with limited human resources and laboratory testing capacity. Therefore, the aim of this study was to determine the prevalence of retinopathy and microalbuminuria and their correlates among hypertensive patients attending medical outpatients' clinic of Mbarara Regional Referral Hospital, a large urban public clinic in western Uganda.

## 2. Materials and Methods

### 2.1. Study Population and Study Design

We conducted a cross-sectional study at the hypertension clinic at the medical outpatients' department of Mbarara Regional Referral Hospital (MRRH), a tertiary facility in the southwestern region of Uganda. The hospital is also a teaching facility for Mbarara University Medical School. The clinic is held once weekly and attends to patients referred from lower health centers including district hospitals and also consultations from within Mbarara Hospital. The clinic is the largest facility providing care for hypertensive patients in the southwestern region and receives approximately 70 patients per clinic day.

### 2.2. Inclusion Criteria and Exclusion Criteria

Patients were eligible to participate if they were 18 years and older, had a previous clinician's diagnosis of hypertension defined as seated systolic/diastolic blood pressure of 140/90 mmHg or above [22], and/or were taking medications for hypertension with documented history of high blood pressure. Patients were excluded if they had diabetes. We excluded hypertensive patients with diabetes because the two conditions cause retinopathy and there is also a likelihood of interaction between the two diseases [23]. Diabetes patients were defined as those on pharmacological treatment for diabetes or those whose random blood sugar (RBS) was above 11.1 mmol/l [24, 25]. Patients were also excluded if they had symptoms of urinary tract infection (UTI) and spot urine dipstick features of UTI, specifically presence of leucocytes, nitrites, and blood in urine.

### 2.3. Data Collection Procedures

Patients seeking treatment for hypertension were given information about the study procedures and if they agreed to participate, they were asked to provide written consent. We collected blood to test for random blood glucose (RBS) level and urine samples to test urine for features of UTI using dipsticks. A standardized, pretested questionnaire was administered to all patients who met the inclusion criteria. Data collected included sociodemographic information such as age, gender, address, occupation, and marital status. We also collected medical history data, duration, and treatment for hypertension. Patients were asked which medications they were taking and clinic books and records were checked to ascertain these medications. Patients were then screened for retinopathy by direct fundoscopy. Urine samples were sent to the Clinical and Research Laboratory at Mbarara University for measurement of urinary albumin and creatinine.

### 2.4. Sample Size and Sampling

We used a standard formula [26] for estimating sample size for a binary outcome: **p**(1 − **p**)**∗**(**Z**^2^_1−**α**/2_)/**d**^2^. In the formula, the estimated prevalence of microalbuminuria is 32% in Nigeria [14] and *d* is the margin of error at 5% using a 95% confidence interval and adjusting for 10% refusal rate; we calculated a sample size of 330 patients. We used consecutive sampling to enroll the participants until the required number was completed.

### 2.5. Data Analysis

Data were entered in Epi Info (version 2007) and exported to STATA 12 (College Station, Texas, USA) for analysis. We summarized sociodemographic and clinical characteristics using descriptive statistics. The categorical or binary variables such as gender and smoking status were summarized using proportions and continuous variables like body mass index (BMI) with means and standard deviations. For nonuniform data, we summarized using medians and interquartile ranges instead of means and standard deviations.

The primary outcomes in the analysis were microalbuminuria and retinopathy. First, we analyzed retinopathy separately and then a combination of the two outcomes, collectively called target organ damage. A participant was considered to have target organ damage (TOD) if they had either one or a combination of both retinopathy and microalbuminuria. Using logistic regression analysis, we explored the association of hypertensive retinopathy and microalbuminuria with predictor variables like gender, smoking status, and duration of hypertension. In the univariate analysis, variables with a *p* value < 0.25 were chosen for inclusion in the multivariate model as important confounding factors. Odds ratios for the association and 95% confidence intervals were reported. Crude and adjusted odds ratio were reported. Results from statistical tests were considered statistically significant if the *p* value was less than 0.05.

### 2.6. Ethical Considerations

All participants provided individual and informed consent. All study procedures were clearly explained to the participants in the language of their choice. Participants who were unable to write offered a thumbprint instead of signature. Patients were assured of their entitlement to standard care even when they chose not to participate or even withdraw from the study after providing consent. Laboratory results and fundoscopy findings were shared with the caregivers to ensure that the participants received adequate management. Data were anonymous and kept in a locked cabinet and were only accessible to study staff. The study received approval from the Research Ethics Committee of Mbarara University of Science and Technology (12/03-13).

## 3. Results

### 3.1. Baseline Characteristics

We screened 400 patients for eligibility and 66 were excluded because they were known diabetics or had RBS > 11.1 mmol/l (*n* = 33), had features of UTI (*n* = 20), had missing urine albumin results (*n* = 7), declined to participate (*n* = 4), or failed to provide urine samples for analysis (*n* = 2). Data was available for analysis for 334 patients. 208 (62.3%) of the patients were female, the median age was 55 years (range: 55 years), and the majority were from Mbarara district. The details of the sociodemographic characteristics are shown in Table 1.

### 3.2. Prevalence of Retinopathy and Microalbuminuria

Of the 334 patients, 223 had retinopathy of different grades, giving overall prevalence of 66.8% (95% CI: 58.6–76.5). The majority of patients with retinopathy were graded as stage 1. Stage 1 hypertensive retinopathy was detected in 90 (40.4%) patients, stage 2 in 81 (36.3%), stage 3 in 50 (22.4%), and stage 4 in only 2 (0.9%) patients.

About 35%  (*n* = 118) of the patients had normoalbuminuria. The prevalence of microalbuminuria (ACR: 30–300 mg/g) was 59.3% (95% CI: 50.1–72.2). The remaining patients (*n* = 18 or 5.4%) had macroalbuminuria (also known as proteinuria)

### 3.3. Factors Associated with Retinopathy

We conducted univariate and multivariable logistic regression analysis to determine the factors associated with retinopathy and the results are indicated in Table 2. In the univariate analysis, the factors associated with retinopathy were duration of hypertension diagnosis greater than five years, having systolic BP greater than 140 mmHg, being overweight (BMI > 25), and being on treatment with beta-blockers, calcium channel blockers, or diuretics. In the multivariate logistic regression analysis, the factors independently associated with retinopathy were more than 5-year duration of hypertension, uncontrolled systolic blood pressure (SPB > 140), and being treated with beta-blockers, calcium channel blockers, or diuretics.

**Figure 1:**
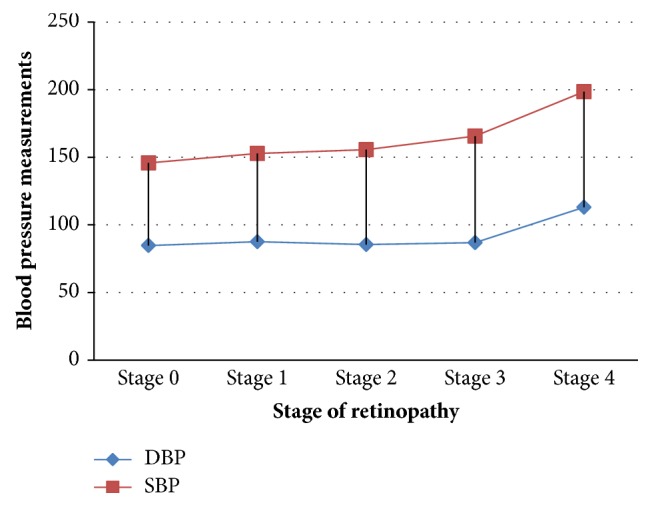
Mean blood pressure measurement by stage of retinopathy.

We assessed the systolic and diastolic blood pressure within each stage of retinopathy. The results are shown in Figure 1. The data show that the mean diastolic blood pressure increased from 84.7 mmHg in stage 0 of retinopathy (no retinopathy) to 113 mmHg among patients in stage 4. Similarly, systolic blood pressure increased from 145.8 mmHg among patients in stage 0 to 198.5 mmHg among patients in stage 4.

### 3.4. Association between Retinopathy and Microalbuminuria

149 (44.6%) of all study participants had both retinopathy and microalbuminuria; 74 (22.2%) had retinopathy but had normal urine albumin. 67 (20.1%) had no retinopathy but had microalbuminuria with proteinuria and 44 (13.1%) had normal urine albumin and no retinopathy. A chi-square test did not show a significant association between retinopathy and microalbuminuria (chi-square 1 degree of freedom = 1.35, *p* = 0.25).

### 3.5. Factors Associated with Target Organ Damage (TOD)

The study participants had TOD if they had either retinopathy or albuminuria or both. The results are summarized in Table 3. Patients older than 65 years had increased odds of TOD and this was significant in the univariate but not in the multivariate analysis. A BMI of less than 25 kg/m^2^ was protective of TOD in the univariate analysis but this effect diminished in the multiple regression analysis. Similarly, patients with hypertension for duration of over 5 years had increased odds for TOD but this effect was dampened in the multivariable analysis.

The only factors that remained significant in the multivariable analysis were high systolic blood pressure and use of beta-blockers. Patients with a systolic blood pressure equal to or greater than 140 had a 2.7-fold increase in the odds of TOD compared to those with systolic blood pressure below 140 (OR = 2.7, 95% CI: 1.29–5.93). Patients who used beta-blockers had a 2.1-fold increase in the odds of TOD (OR = 2.16, 95% CI: 1.05–4.44) compared to those not using beta-blockers.

## 4. Discussion

Our study among an African hypertensive and nondiabetic population has shown that prevalence of microalbuminuria and that of retinopathy are very high. Patients with poorly controlled blood pressure were more likely to have target organ damage, and so did patients with long-standing hypertension. These findings are comparable with other studies on the African continent but some differences emerge on and outside the continent.

The prevalence of microalbuminuria at 57.5% in our study is higher than that found in similar studies in Uganda [15]. The prevalence of microalbuminuria is quite variable across the African continent with lower prevalence of 32% in a Nigerian study [14] but was much higher in a similar study in Morocco [27]. The differences could be explained by the different patient characteristics, because they enrolled newly diagnosed hypertensive patients, while the present study enrolled already known hypertensive patients. This also means that the patients in our study had lived longer with the hypertension and therefore were likely to have more vascular damage as manifested by the higher prevalence of microalbuminuria. However, recent studies suggest that the prevalence can be higher than 60%, indicating the widespread nature of renal complications [28].

In the Microalbuminuria A Genoa Investigation on Complications (MAGIC) study [29], which was done in Italy, the prevalence of microalbuminuria was 6.7%. This observation suggests that prevalence of microalbuminuria among African patients may be much higher than those in the western world. We should note that the MAGIC study excluded patients with severe hypertension and those with obesity and renal disease, while our study did not. However, prevalence of microalbuminuria may still be higher in the African continent because majority of hypertensive patients have poorly controlled blood pressure, rendering them at higher risk for kidney damage. Recent Ugandan [30] and Kenyan studies [11, 31] showed that less than 50% of patients had adequate blood pressure control.

In our study, no medications were associated with microalbuminuria. This does not agree with findings from other studies. One such study in an African setting [32] showed that treatment with calcium channel blockers was associated with microalbuminuria, although our study did not show an association. The questions on medications require a separate study as our study was not powered to answer these questions. Secondly, these studies will require comprehensive inquiry on medication history including adherence to medicines.

The present study found 66.8% prevalence of hypertensive retinopathy in outpatients on treatment for hypertension in MRRH. The findings on retinopathy are fairly homogenous to those found in other African studies. In an Ugandan study, the prevalence of hypertensive retinopathy among patients on treatment for hypertension in Mulago Hospital, a national referral hospital, was 70.2%. This is also comparable to findings in a study done in Nigeria, which found 71% prevalence of hypertensive retinopathy [14]. The results are comparable because of similar characteristics of the populations studied and the methods used. The patients in all the above studies were black Africans and had uncontrolled blood pressure.

Outside the African continent, comparable results were also found in Turkey, where the prevalence of hypertensive retinopathy was 66.3% [33]. Similar to observations in our study, the lower grades of hypertensive retinopathy were most prevalent. The high prevalence of retinopathy in this non-African population is also probably explained by the older age of patients studied. In our study, we enrolled patients as old as 80 years and age is a well-known risk factor for increased prevalence of hypertensive retinopathy even in persons without hypertension [34].

Factors associated with retinopathy were high systolic BP, being hypertensive for more than five years, high BMI, and being on treatment with *β*-blockers, calcium channel blockers, and diuretics. The correlates of multiple target organ damage (retinopathy, microalbuminuria, and proteinuria) included uncontrolled hypertension and taking beta-blockers. Duration of hypertension was not a significant predictor. Surprisingly, the use of ACEIs was not a protective factor. Controlled blood pressure was protective of target organ damage. This is a very important observation because it makes the case for advocating for tight BP control and use of ACEIs in our patients. It has already been shown in many other studies that ACEIs reduce microalbuminuria [7]. Potentially, the explanation of the association of beta-blockers with target organ damage is that these are usually prescribed to patients with resistant hypertension or those for whom ACEIs are contraindicated. Our study was not powered to answer questions on drug efficacy.

Our study has some limitations. Firstly, we did not have a fundus camera. This would have enabled recording of retinal photographs and their subsequent interpretation by independent assessors to improve accuracy of diagnosis. Secondly, some of our patients were elderly and, at that age, retinal changes may begin to manifest (probably due to generalized atherosclerosis and not necessarily due to hypertension). Thirdly, we did not measure renal function due to limited funds. Lastly, we were unable to perform urine culture and lipid profiles. These variables may be potential confounders in this study because infections and hyperlipidemia have an effect on hypertensive retinopathy and microalbuminuria.

In conclusion, the prevalence of hypertensive retinopathy and microalbuminuria in hypertensive patients attending the medical outpatients' clinic of a large urban public hospital in Uganda is high. On the basis of our findings, we recommend rigorous blood pressure control to maintain systolic BP < 140 mmHg. Clinicians should endeavor to conduct fundoscopy and measure urine albumin of all hypertensive patients who are on treatment with *β*-blockers and those with systolic BP > 140 mmHg despite taking antihypertensive treatment.

## Figures and Tables

**Table 1:** Baseline characteristics of the study participants enrolled at hypertension clinic, Mbarara Hospital (*n* = 334).

Characteristic	*n* = 334
Characteristics	
Age, median (range)	55 (25–87)
Female, *n* (%)	208 (62.3)

District of residence, *n* (%)	
Mbarara	177 (53.0)
Isingiro	63 (18.9)
Bushenyi	40 (12.0)
Ntungamo	14 (4.2)
Others	40 (12.0)

Occupation, *n* (%)	
Peasant	112 (33.5)
Self-employed	130 (38.9)
Professional	11 (3.3)

Education, *n* (%)	
None	99 (29.6)
Primary	150 (44.9)
Secondary	56 (16.8)
Tertiary	29 (8.7)

Clinical	
Current smoker, *n* (%)	64 (19.2)
Duration of hypertension (years), median (range)	4.75 (1–12)
History of stroke/TIA, *n* (%)	12 (3.59)
Body mass index, mean (±SD)	28.19 (17.9)
Systolic blood pressure, mean (±SD)	153 (22.2)
Diastolic blood pressure, mean (±SD)	86 (12.9)
Fasting blood sugar, mean (±SD)	6.16 (1.6)
Urine albumin concentration, mean (±SD)	3904.4 (47.5)
Urine creatinine, mean (±SD)	62.85 (5.2)
Urinary albumin-creatinine ratio, mean (±SD)	87.7 (14.1)

Medication	
ACEI/ARB, *n* (%)	103 (30.8)
Blockers, *n* (%)	145 (43.4)
Calcium channel blockers, *n* (%)	232 (69.5)
Diuretics, *n* (%)	199 (43.4)

**Table 2:** Logistic regression analysis of factors associated with retinopathy at Mbarara Regional Referral Hospital, Mbarara, Uganda (*n* = 334 patients).

Explanatory variables (characteristics)	Univariate analysis			Multivariate analysis		
	OR	95% CI	*p* value	OR	95% CI	*p* value
Sex						
Male	1.0					
Female	0.79	0.49–1.26	0.323			

Age						
Age < 65 years	1.0			1.0		
Age > 65 years	2.52	1.63–3.94	0.0001	3.75	2.07–5.39	0.0001

Smoking history						
Current smoker						
Nonsmoker	1.34	0.74–2.45	0.336			

Exercising						
Regular exercise						
Irregular/no exercise	0.95	0.55–1.62	0.837			

Duration of hypertension diagnosis						
≤5 years						
>5 years	4.49	2.68–7.49	0.0001	3.73	2.12–6.57	0.0001

SBP						
≥140 mmHg						
<140 mmHg	2.78	1.69–4.55	0.0001	3.53	1.99–6.24	0.0001

DBP						
≥90 mmHg						
<90 mmHg	1.11	0.69–1.79	0.65			

BMI						
≥25 Kg/m^2^	1.0					
<25 Kg/m^2^	0.76	0.48–1.19	0.230	0.92	0.54–1.56	0.75

ACEI						
Taking ACEIs						
Not taking ACEIs	1.15	0.70–1.89	0.58			

*β*-blocker						
Not Taking *β*-blockers	1.0					
Taking *β*-blockers	1.88	1.17–3.02	0.009	3.05	1.68–5.53	0.0001

Calcium blocker						
Use calcium blockers						
No calcium blockers	1.65	1.02–2.68	0.042	2.16	1.19–3.90	0.011

Diuretic						
Use diuretics						
No diuretics	2.91	1.82–4.66	0.0001	2.91	1.68–5.04	0.0001

Microalbuminuria (ACR 30–300 mg/g)	1.23	0.78–1.96	0.369	1.39	0.81–2.40	0.225

Proteinuria (ACR > 300 mg/g)	1.32	0.82–2.12	0.246			

*Note.* Variables with a *p* value less than 0.25 were then included in the multivariate model.

**Table 3:** Logistic regression analysis of factors associated with target organ damage.

Explanatory variables (characteristics)	Univariate analysis			Multivariate analysis		
	OR	95% CI	*p* value	OR	95% CI	*p* value
Sex						
Male	1.0					
Female	0.77	0.41–1.46	0.42			

Age						
≤65 years	1.0			1.0		
>65 years	2.11	1.76–3.87	0.001	1.65	0.93–3.36	0.15

Smoking history						
Current smoker						
Nonsmoker	1.29	0.55–3.05	0.56			

Exercising						
Regular exercise						
Irregular/no exercise	1.17	0.53–2.55	0.69			

Duration of hypertension diagnosis						
≤5 years	1.0			1.0		
>5 years	2.03	1.03–3.98	0.04	1.74	0.85–3.59	0.13

SBP						
<140 mmHg	1.0			1.0		
≥140 mmHg	2.92	1.53–5.58	0.001	2.76	1.29–5.93	0.01

DBP						
<90 mmHg	1.0			1.0		
≥90 mmHg	1.73	0.85–3.49	0.13	1.22	0.53–2.81	0.64

BMI						
≥25 Kg/m^2^						
<25 Kg/m^2^	0.49	0.25–0.96	0.039	0.59	0.28–1.14	0.11

ACEI						
Not taking ACEIs						
Taking ACEIs	1.39	0.67–2.88	0.37			

*β*-blocker						
No beta-blockers						
Beta-blockers	1.99	1.0–3.96	0.05	2.16	1.05–4.44	0.04

Calcium blocker						
Calcium blockers						
No calcium blockers	1.07	0.54–2.12	0.84			

Diuretic						
Taking diuretics						
Not taking diuretics	1.74	0.92–3.29	0.088	1.60	0.80–3.20	0.18

*Note.* Variables with a *p* value less than 0.25 were then included in the multivariate model.

## Data Availability

Data are available upon request to eligible applicants.

## Additional Points

Our study in an African hypertensive population indicates that prevalence of microalbuminuria and retinopathy, known predictors of cardiovascular disease, is high. Clinicians should aim to control blood pressure to reduce the occurrence of these complications.
